# Antioxidant and Antibacterial Potential of *Passiflora edulis* (Passion Fruit) at Three Ripening Stages for Waste Valorization

**DOI:** 10.3390/molecules30173454

**Published:** 2025-08-22

**Authors:** Mariela Quirós-Cubillo, Sandra Valdés-Díaz, Juan Oviedo-Quirós, Víctor Álvarez-Valverde, Randall Syedd-León

**Affiliations:** 1 Natural and Exact Sciences School, Universidad Estatal a Distancia, Mercedes de Montes de Oca, San José 474-2050, Costa Rica; quiros.cm29@gmail.com; 2 Chemistry School, Universidad Nacional of Costa Rica, Heredia 86-3000, Costa Rica; sandra.valdes.diaz@una.ac.cr; 3 Department of Agricultural Sciences, Costa Rican Ministry of Agriculture, San José 10094-1000, Costa Rica; 4 Phytochemistry Laboratory, Department of Chemistry, Universidad Nacional of Costa Rica, Heredia 86-3000, Costa Rica; victor.alvarez.valverde@una.ac.cr

**Keywords:** *Passiflora edulis*, antioxidant activity, antibacterial potential, ripening stages, nutraceutical applications

## Abstract

This study evaluated the antioxidant and antibacterial potential of *Passiflora edulis* (passion fruit) at three ripening stages—intermediate, ripe, and overripe—to determine the optimal consumption time based on the presence of secondary metabolites (polyphenols, alkaloids, and anthocyanins). Fruits from Costa Rica, including pulp and peel, were analyzed. Qualitative assays (Dragendorff, Mayer, Lieberman Burchard, Ferric Chloride, and Shinoda) and quantitative analyses using Folin–Ciocalteu (total polyphenols), ORAC (antioxidant activity), and Kirby–Bauer (antibacterial activity) methods were conducted. Acetone–water (7:3) was the most effective solvent, with three extractions yielding optimal results. Peels contained significantly higher polyphenols (7.2 ± 0.1 mg GAE/g d.w.) and antioxidant activity (2403 ± 519 µmol TE/g d.w.) than pulps. Anthocyanins were abundant in both, while antibacterial activity was more effective in peels, inhibiting Gram-positive bacteria with 25% relative inhibition, but showing no activity against Gram-negative strains. These findings highlight passion fruit peel as a rich source of bioactive compounds with strong antioxidant and antibacterial properties, particularly in intermediate and overripe stages, supporting its potential use in the development of functional ingredients for nutraceutical applications and promoting sustainable waste management.

## 1. Introduction

Passion fruit (*Passiflora edulis*), a member of the *Passifloraceae* family, is a tropical fruit native to South America, with Brazil being considered its primary center of origin. This species includes two main varieties, the yellow passion fruit (*P. edulis f. flavicarpa*) and the purple passion fruit (*P. edulis f. edulis*), which are both widely distributed across Spanish-speaking countries in the Americas. *P. edulis* exhibits significant phenotypic variability, particularly in fruit size and color. It is commonly consumed fresh, but its distinctive acidic flavor and appealing organoleptic properties make it a key ingredient in various processed products, including beverages and desserts [1].

Recent studies have demonstrated that *Passiflora edulis* is rich in secondary metabolites such as polyphenols, flavonoids, and anthocyanins, which are responsible for its potent antioxidant and antibacterial activities [2]. The accumulation of these bioactive compounds is known to vary throughout the fruit’s development and ripening, with earlier ripening stages showing higher levels of phenolic compounds associated with enhanced antioxidant capacity [3]. Passion fruit peel, often discarded as agricultural waste, has gained scientific attention for its considerable concentration of bioactive constituents, supporting its revalorization in nutraceutical and pharmaceutical applications [4]. Despite this potential, comparative studies on the antioxidant and antibacterial profiles of both pulp and peel across different ripening stages remain limited, emphasizing the relevance of the present research. Furthermore, exploring waste valorization strategies aligns with current trends in sustainable agriculture and circular economy initiatives [5].

Agriculture in Costa Rica plays an essential role in the country’s socio-economic development, accounting for a significant portion of both its economic activity and rural employment [6]. In recent years, the sector has increasingly adopted circular economy models that emphasize value-added production and consumption. These models aim not only to reduce food waste but also to foster its reuse, creating new opportunities for sustainability [7]. Such advancements have spurred the study and implementation of biochemical industrial processes [8], which target more sustainable markets [9] with improved nutritional compositions and broader consumer acceptance [10].

Currently, numerous governmental initiatives are being promoted within the framework of more sustainable practices. These actions focus on evaluating the life cycle of consumer products, particularly in terms of balancing the health benefits they provide to humans [11] with their environmental impact, specifically the waste generated during various production stages—from the initial producers to final consumers [12]. This balanced approach aims to encourage environmentally conscious practices within industries and among consumers alike.

National crops like passion fruit are a key example of agricultural products with both exceptional organoleptic properties—such as a distinct aroma and high acidity—and considerable nutritional value. Rich in vitamins A and C, as well as other essential minerals, passion fruit has gained recognition as a functional food [13,14,15]. Its annual exports, totaling 432 tons in 2018, are growing steadily as its consumption continues to rise. The fruit is primarily enjoyed fresh in juices, beverages, and concentrates, and it also serves as a raw material for various by-products such as sauces, dressings, and jams in the food industry.

While the pulp is the most commonly utilized part of the passion fruit, the peel and endocarp are typically discarded as waste in landfills [16]. However, the natural decomposition of these parts is slow and inefficient, which makes them unsuitable as effective fertilizers. Interestingly, their biochemical components may exhibit resistance or inhibitory effects against the microorganisms involved in decomposition processes [17]. This suggests that these parts may hold untapped potential for other applications, particularly in the context of the circular economy, which seeks to find value in products and materials that would otherwise be discarded.

The biological properties of plants, including those found in passion fruit, are often attributed to the production of secondary metabolites. These compounds are synthesized by plants in response to environmental stress, such as exposure to pathogens or extreme weather conditions [18]. Among these metabolites, polyphenols are particularly significant. Found in abundance in passion fruit, polyphenols have been widely studied for their pharmacological properties, including strong antioxidant effects and potential disease-preventing capabilities [19,20,21]. These findings highlight the need to further explore the antioxidant and other bioactive potential of passion fruit, particularly in its various stages of maturation.

Thus, the current study aimed to evaluate and compare the antioxidant and antibacterial activities of *Passiflora edulis* pulp and peel across three distinct ripening stages. Understanding the variation in metabolite profiles during fruit development may enable a better utilization of passion fruit by-products, support waste valorization strategies, and enhance the production of functional ingredients for nutraceutical applications.

## 2. Results and Discussion

### 2.1. Qualitative Determination of Secondary Metabolites

The composition of secondary metabolites in *P. edulis* is highly diverse, as the presence of major groups in different qualitative proportions was identified (Table 1). As previously mentioned, the results are shown for both peel (C) and pulp (P) at the three ripening stages under study (labeled M1, M2, and M3). This interpretation should be viewed qualitatively, in which the colorimetric scale of color intensity is related to its relative abundance.

In general, the presence of quinones and cardiotonic glycosides was not detected. The groups of alkaloids and tannins, as well as free phenols, were found in barely perceptible amounts, meaning they were below the detection limit of the tests performed. On the other hand, it is particularly noteworthy that coumarins and anthocyanins are relatively abundant and present in both the fruit and the peel, without differentiation by ripening stage.

Although the qualitative detection methods employed in this study (colorimetric assays) provide valuable preliminary information on the presence of major secondary metabolites, they lack the specificity and sensitivity of advanced chromatographic techniques such as HPLC-MS or UPLC-MS/MS. Future studies should incorporate these methods to achieve precise profiling of individual bioactive compounds. Nevertheless, the use of qualitative assays remains justified in preliminary screenings, particularly in contexts of limited laboratory resources or when the goal is to explore general metabolite patterns rather than to characterize individual molecules [22,23].

The results obtained are consistent with previous findings reporting positive responses for anthocyanins, coumarins, and flavonoids in passion fruit peel [24]. Furthermore, the abundant presence of anthocyanins, coumarins, and flavonoids provides evidence of the antioxidant and antibiotic capacity of both peel and pulp, as these groups are closely associated with phenolic content and biological activity.

The high abundance of anthocyanins, flavonoids, and coumarins detected in the peel correlates with the elevated antioxidant capacity and antibacterial activity observed in subsequent assays. Anthocyanins contribute to free radical scavenging and anti-inflammatory effects [25], while flavonoids are recognized for their ability to disrupt bacterial membranes and inhibit microbial growth [26]. Similarly, coumarins have been linked to antimicrobial mechanisms involving the inhibition of bacterial DNA replication and oxidative stress modulation [27]. This suggests that the secondary metabolite composition plays a pivotal role in the bioactivities recorded during the maturation process of the fruit.

### 2.2. Determination of the Best Solvent and Optimal Number of Extractions

The determination of total polyphenol content (TPC) requires finding optimal extraction conditions, which vary depending on the food matrix. Since phenolic compounds are polar in nature, several solvent systems of similar polarity were tested (S1: water; S2: acetone–water (7:3); S3: ethanol–water (7:3); and S4: 95% ethanol). A careful selection of the extraction solvent, combined with the appropriate number of successive extractions, prevents the underestimation of phenolic content and improves reproducibility. This assay was conducted on the peel, as it is the part of the fruit typically discarded during consumption and, according to previous results, contains the greatest diversity of secondary metabolites.

**Figure 1:**
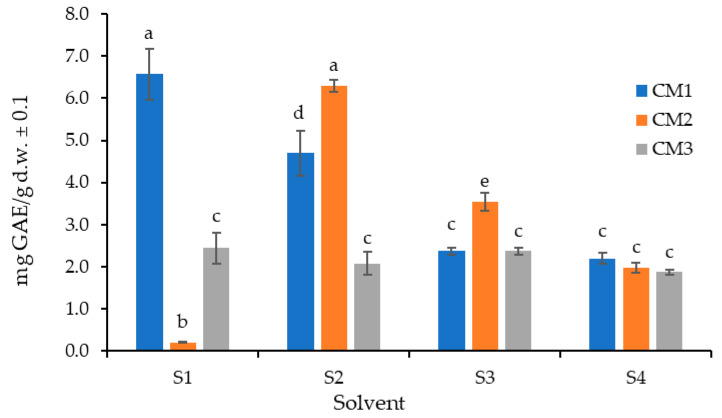
Comparison of total polyphenol extraction from *P. edulis* peel (C) using the Folin–Ciocalteu method with different extraction solvents (S1: water; S2: acetone–water (7:3); S3: ethanol–water (7:3); and S4: 95% ethanol) at three ripening stages (M1: intermediate; M2: ripe; and M3: overripe). Different letters indicate statistically significant differences according to Dunn’s post hoc test with Bonferroni correction (Kruskal–Wallis, *p* < 0.05).

Figure 1 presents a bar graph showing the amount of polyphenols extracted from the peel using the four solvents at three ripening stages. When considering the results by ripening stage, in M1, the best solvent was S1; in M2, the best solvent was S2 (*p* < 0.05); and in M3, all solvents exhibited statistically the same extraction efficiency. Overall, when considering all three ripening stages collectively, S2 (acetone–water, 7:3) was the most effective solvent and was therefore selected for further analyses.

Previous studies support the use of acetone–water (7:3) for optimal polyphenol yield in *Byrsonima crassifolia* (2.9 ± 0.3 mg GAE/g dry matter) [28], as well as in brewer’s grain extracts (0.16 ± 0.01 mg GAE/L for total polyphenol content and 0.06 ± 0.01 mg TE/L for antioxidant activity) [29]. Additionally, this solvent system has been reported for polyphenol extraction and antioxidant activity evaluation in *Eucalyptus camaldulensis* [30,31] and for total polyphenol extraction in yellow passion fruit (*Passiflora edulis f. flavicarpa*) (2.05 ± 0.04 g GAE/kg) [31].

Similarly, phenolic content obtained with other solvent mixtures such as ethanol–water has been reported to yield lower extraction efficiencies in passion fruit peels and pulps [32,33,34].

**Figure 2:**
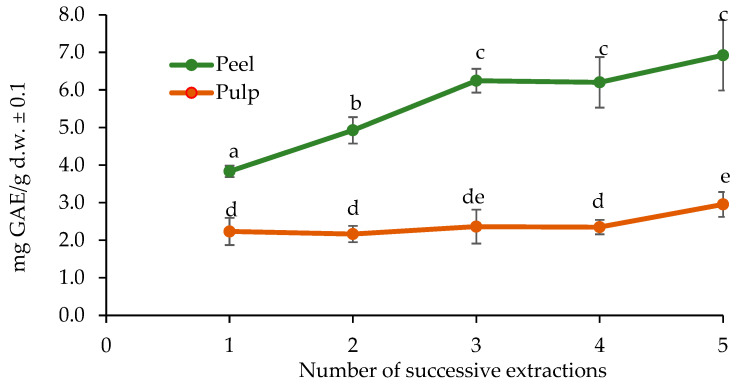
Comparison of total polyphenol extraction (TPC) from the peel and pulp of *P. edulis* in the intermediate ripening stage (CM1) using the Folin–Ciocalteu method with different numbers of successive extractions. Different letters indicate statistically significant differences according to Dunn’s post hoc test with Bonferroni correction (Kruskal–Wallis, *p* < 0.05).

On the other hand, Figure 2 shows that the determination of the optimal number of extractions indicated no significant difference in the TPC in the pulp between extractions 1 and 4, as well as between 3 and 5. However, in the peel, a significant difference was observed between extractions 1, 2, and 3, but not between 3, 4, and 5.

Thus, three successive extractions were determined as the most suitable protocol to maximize polyphenol recovery without unnecessarily increasing processing time and resources. This finding aligns with previous studies on phenolic extraction optimization [35,36,37]. Optimizing both the solvent system and the number of extractions ensures the maximum recovery of phenolic compounds and antioxidant activities while minimizing operational efforts.

### 2.3. Total Polyphenol Determination by Folin–Ciocalteu

**Figure 3:**
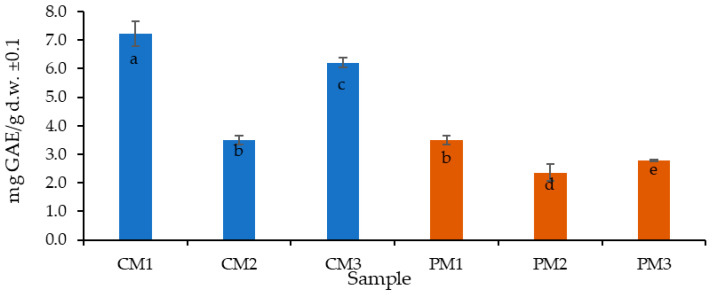
Distribution of total polyphenol content (TPC) in *P. edulis* peel (C) and pulp (P) at three ripening stages (M1: intermediate; M2: ripe; and M3: overripe). Different letters indicate statistically significant differences according to Dunn’s post hoc test with Bonferroni correction (Kruskal–Wallis, *p* < 0.05).

The TPC results are reported as gallic acid equivalents per gram of dry sample, which is the most common form in food samples, facilitating comparisons. Figure 3 presents the results for the peel (C) and pulp (P) at the three ripening stages (M1, M2, and M3).

The TPC was higher in the peel (5.7 mg GAE/g d.w.) than in the pulp (2.9 mg GAE/g d.w.) by almost twice as much. It is important to highlight that the peel, typically discarded as waste, holds significant potential for bioactive compound recovery and sustainable valorization.

Additionally, the highest TPC was found in the intermediately ripe peel (CM1) with 7.2 ± 0.1 mg GAE/g d.w. (*p* < 0.05), followed by the overripe peel (CM3) with 6.2 ± 0.2 mg GAE/g d.w., and the pulp at the intermediate ripening stage (PM1) with 3.5 ± 0.2 mg GAE/g d.w. The ripening stage appears to have an inversely proportional effect on the pulp, meaning that as ripening progresses, the TPC decreases. A similar but less pronounced trend was observed in the peel.

This behavior can be explained by the ripening process, during which starch is rapidly hydrolyzed into glucose and fructose, tannins are reduced, and pH increases, potentially affecting phenolic compound stability [38].

In comparative terms, a study on the by-products (peel, pulp, and seeds) of *P. edulis* L. cv. *flavicarpa* reported values of 2.46 mg GAE/g d.w. [39], similar to those obtained for PM2 and PM3 in the present study. Other reports have indicated 74.61 mg GAE/mL in pulp extracts [33], as well as 6.35 ± 0.03 mg GAE/g d.w. in *P. mollissima* and 10.18 ± 0.14 mg GAE/g d.w. in *P. tarminiana* (“Curuba quiteña” or banana passion fruit) [40], which are more comparable to the results observed for CM1 and CM3. Moreover, values for passion fruit seeds reported in other studies (0.32–0.39 g GAE/g d.w.) [41,42] are considerably lower compared to the peel values obtained here.

**Figure 4:**
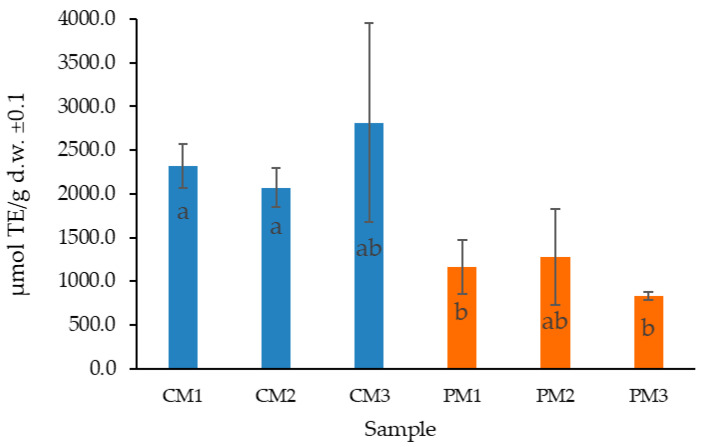
Distribution of antioxidant content in *P. edulis* peel (C) and pulp (P) at three ripening stages (M1: intermediate; M2: ripe; and M3: overripe), determined by the ORAC method. Different letters indicate statistically significant differences according to Dunn’s post hoc test with Bonferroni correction (Kruskal–Wallis, *p* < 0.05).

The relatively high standard deviation values observed in certain samples (e.g., in Figure 4), particularly in CM3, can be attributed to biological variability among fruits, heterogeneity in the distribution of secondary metabolites within tissues, and the inherent challenges in defining a standardized maturity parameter [43]. Such variability is commonly reported in biological matrices and should be carefully considered when interpreting quantitative results [44]. These findings further confirm that the significant differences observed between peel and pulp are the result of multiple interacting factors, including fruit ripeness, varietal characteristics, soil composition, genetic background, and variations in processing and extraction methodologies [45,46]

Overall, the results confirm that peel-derived extracts are significantly richer in polyphenolic compounds compared to pulp, supporting their potential as a valuable by-product for nutraceutical applications.

### 2.4. Determination of Antioxidant Activity by ORAC

The antioxidant activity (AA) of the extracts was determined using the oxygen radical absorbance capacity (ORAC) assay, employing Trolox as the reference antioxidant standard. The results are expressed as micromoles of Trolox equivalents per gram of dry weight (µmol TE/g d.w.), reflecting the synergistic interaction of all antioxidants present in the sample.

Consistent with the TPC findings, the peel extracts (CM1, CM2, and CM3) exhibited significantly higher antioxidant activities compared to pulp extracts (PM1, PM2, and PM3) (*p* < 0.05). On average, the antioxidant activity in the peel samples was 2403 ± 519 µmol TE/g d.w., while in the pulp, it was 1092 ± 252 µmol TE/g d.w.

No significant differences were observed between the different ripening stages in either peel or pulp (*p* > 0.05), suggesting that the antioxidant potential remained relatively stable throughout fruit maturation. This stability indicates that passion fruit peel waste could be valorized regardless of the stage at which fruits are discarded, enhancing its applicability in functional foods or nutraceutical products.

Few studies have reported antioxidant activity in *P. edulis* using the ORAC method with acetone–water extracts. The antioxidant capacity obtained in this study for peel samples notably exceeded values reported for other plant materials, including ethanol-based extracts of *P. mollissima* pulp (207.6 ± 1.9 µmol TE/g d.w.) [47] and aqueous or ethanolic extracts from different passion fruit species, which often exhibit lower activities.

Previous reports have indicated ORAC values in ethanolic peel extracts of *P. edulis* Sims around 494 ± 22 µmol TE/g d.w., and in seeds, values of 1428 ± 30 µmol TE/g d.w. are reported, which are substantially lower than those obtained here [48]. Similarly, values of 68.6 ± 0.1 µmol TE/g d.w. have been reported for peel flour obtained from *P. edulis* Sims f. *flavicarpa* [49].

The results obtained are likely explained by the optimization of extraction conditions employed in this study (choice of solvent, an acetone–water ratio of 7:3, and the use of three successive extractions), which maximized the recovery of phenolic and antioxidant compounds. In addition, differences in passion fruit genotype and environmental conditions in Costa Rica could contribute to the superior antioxidant activity measured.

**Figure 5:**
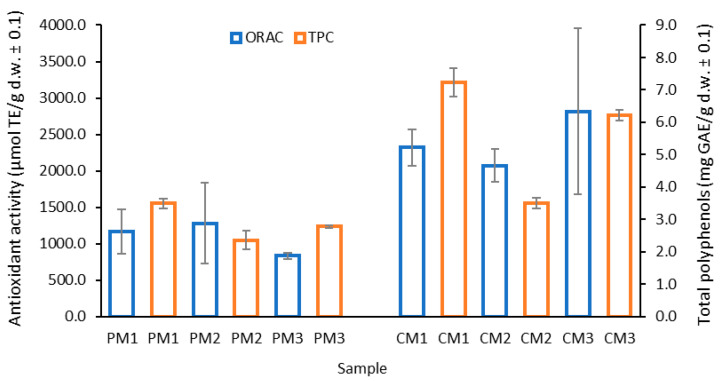
Correlation between total polyphenol content (TPC) and antioxidant potential (ORAC) for peel (C) and pulp (P) samples of *P. edulis*.

Furthermore, the correlation between TPC and antioxidant activity was evaluated using Spearman’s correlation test, yielding a positive relationship (r^2^ = 0.67; *p* < 0.05), as shown in Figure 5. The strength of this relationship is not extremely high, but it is still statistically significant. This finding aligns with previous studies on various food matrices [44].

This positive correlation confirms that the polyphenols present in the passion fruit extracts play a major role in their antioxidant potential, although other classes of antioxidants (such as carotenoids and vitamin C) may also contribute synergistically to the overall activity.

### 2.5. Bacterial Susceptibility Testing

**Figure 6:**
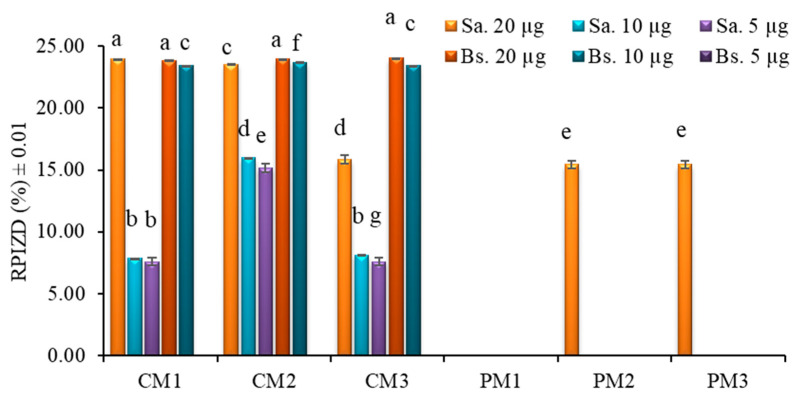
Distribution of antibiotic activity against *S. aureus* (Sa.) and *B. subtilis* (Bs.) relative to the positive control (gentamicin 10 µg) in the peel (C) and pulp (P) of *P. edulis* at three ripening stages (M1: intermediate; M2: ripe; and M3: overripe) and at three different concentrations, determined using the Kirby–Bauer method. Different letters indicate statistically significant differences according to Dunn’s post hoc test with Bonferroni correction (Kruskal–Wallis, *p* < 0.05). RPIZD: relative percentage of inhibition zone diameter.

Extracts from *P. edulis* peel (C) and pulp (P) at three ripening stages (M1, M2, and M3) and at three concentrations (20 µg, 10 µg, and 5 µg) were tested against selected pathogenic microorganisms using the Kirby–Bauer disk diffusion method (Figure 6).

The results showed no visible inhibition zones for Gram-negative bacterial cultures (*Escherichia coli* and *Pseudomonas fluorescens*) at any concentration tested. In contrast, Gram-positive bacteria (*Staphylococcus aureus* (Sa.) and *Bacillus subtilis* (Bs.)) exhibited measurable inhibition zones, particularly in peel extracts.

At the highest tested concentration (20 µg), peel extracts (CM1, CM2, and CM3) produced inhibition zones corresponding to approximately 25% of the positive control’s (gentamicin 10 µg) relative inhibition zone diameter (RPIZD). Pulp extracts (PM2 and PM3) showed lower inhibition activity, reaching relative inhibition percentages below 18%.

**Figure 7:**
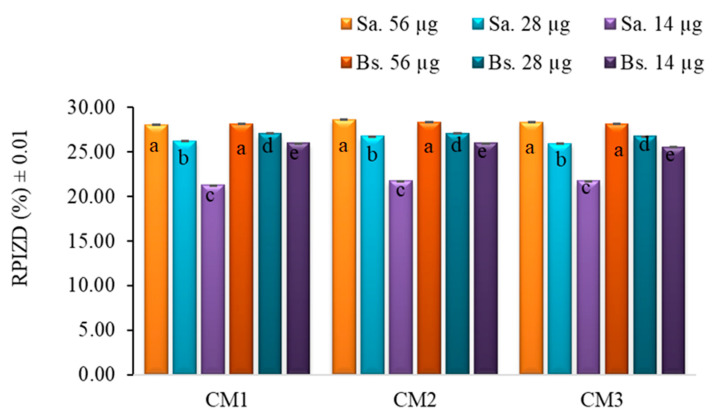
Distribution of the antibiotic activity observed in *S. aureus* (Sa.) and *B. subtilis* (Bs.) relative to the positive control (gentamicin 10 µg), in extracts from the peel (C) and pulp (P) of *P. edulis* at three ripening stages (M1: intermediate; M2: ripe; and M3: overripe) and at three concentrations, determined by the Kirby–Bauer method. Different letters indicate statistically significant differences according to Dunn’s post hoc test with Bonferroni correction (Kruskal–Wallis, *p* < 0.05). RPIZD: relative percentage of inhibition zone diameter.

To confirm the initial findings, a second antibiogram was performed using peel extracts at higher concentrations (56 µg, 28 µg, and 14 µg). Despite the increased dosage, no antibacterial activity was observed against the Gram-negative strains *E. coli* and *P. fluorescens*. On the other hand, a concentration-dependent increase in antibacterial activity was observed against Gram-positive bacteria (*S. aureus* and *B. subtilis*), reaching up to 28% RPIZD relative to the positive control (gentamicin 10 µg) at the highest concentration tested. No statistically significant differences were found between the two Gram-positive strains at the same extract concentration (Figure 7).

The lack of activity against Gram-negative strains is likely explained by the presence of an outer membrane composed of lipopolysaccharides, which acts as a selective permeability barrier and protects the underlying peptidoglycan layer from hydrophilic and many hydrophobic substances [50]. In contrast, Gram-positive bacteria possess a thicker, but more permeable, peptidoglycan wall, making them more susceptible to the action of phenolic compounds, flavonoids, and other bioactive molecules present in passion fruit extracts [51].

In this study, the limited inhibition observed in pulp extracts (PM1, PM2, and PM3) at 20 μg suggests that the concentration used was insufficient to achieve a broader antimicrobial effect. Previous reports have indicated that acetone extracts from passion fruit pulp at 128 mg/mL produced inhibition zones of 15.00 ± 0.81 mm for *S. aureus*, 17.33 ± 0.94 mm for *B. subtilis*, 14.00 ± 0.81 mm for *E. coli*, and 18.66 ± 0.47 mm for *P. aeruginosa* [52]. Similarly, the peel extracts tested at 56 μg yielded inhibition zones of 6.60 ± 0.02 mm for *S. aureus* and 6.10 ± 0.01 mm for *B. subtilis*, which are lower than previously reported values of 8.00 ± 0.00 mm and 11.66 ± 0.47 mm, respectively [52]. These comparisons indicate that the antimicrobial activity of the extracts is dose-dependent and that significantly higher concentrations may be required to achieve inhibition levels comparable to those reported in the literature.

Overall, these results suggest that passion fruit peel extracts, particularly from earlier ripening stages, may serve as a potential source of natural antimicrobial agents against Gram-positive bacteria, although further optimization and fractionation would be necessary to enhance efficacy.

## 3. Materials and Methods

### 3.1. Sample Collection and Treatment

Fruits of *P. edulis* were randomly collected from a commercial farm located in Batán, Limón, Costa Rica (N: 10.093047; W: 83.387119), at an altitude of 35 m above sea level. The sampling was conducted during the main harvest season (March–May) to minimize variability due to seasonal effects.

Seasonal temperature variations between the dry and rainy periods (April to November) are minimal (±3 °C) and are therefore unlikely to significantly affect secondary metabolite production. This assumption is supported by previous studies, which concluded that, in general, seasonality did not directly impact the quantitative production of polyphenols, flavonoids, and rosmarinic acid. Factors such as solar radiation, rather than temperature or precipitation variations, had a clearer influence on the production of phenolic compounds and on the photoprotective and antioxidant activities of both species [53].

Fruit selection was standardized according to external coloration and firmness, and visually classified into three ripening stages:

- M1: Fruit at an early stage of ripening (some green coloration on peel; intermediate maturation);
- M2: Ripe fruit (fully colored peel; optimal for commercialization);
- M3: Overripe fruit (wrinkled or discolored peel; signs of post-climacteric stage).

Samples were taken from different trees across several rows of the plantation to increase representativeness. Pulp (separated from seeds) and peel were isolated manually, frozen at −40 °C for 72 h, lyophilized, and ground with a blade mill to a particle size of 0.75 mm.

### 3.2. Qualitative Determination of Secondary Metabolites

The preliminary qualitative identification of secondary metabolites in *P. edulis* pulp and peel was performed using standard phytochemical screening methods. Alkaloids were detected through Dragendorff’s and Mayer’s tests; triterpenes by the Liebermann–Burchard reaction; saponins by the foam test; tannins and phenols by ferric chloride reaction and gelatin precipitation; flavonoids by the Shinoda reaction; leucoanthocyanins by acid hydrolysis tests; cardiotonic glycosides by the Kedde reaction; quinones by reactions with alcoholic potassium hydroxide; anthocyanins through color changes under acidic and alkaline conditions; and coumarins by UV fluorescence after treatment with potassium hydroxide [54,55,56,57,58,59].

Each assay was conducted in triplicate for both the pulp and peel at different ripening stages. The results were interpreted based on the intensity of the color reaction or precipitation observed and classified on a nominal scale: abundant (+++), moderate (++), scarce (+), doubtful (±), or not detected (nd).

In this study, classical phytochemical screening methods were employed to perform a preliminary qualitative assessment of the main secondary metabolite groups present in *P. edulis* pulp and peel across different ripening stages. These techniques, although not as specific as chromatographic methods such as HPLC or UPLC-MS/MS, are well-recognized tools for the initial identification of broad metabolite classes in plant matrices, particularly in exploratory bioactivity studies [60].

### 3.3. Determination of the Best Solvent and Optimal Number of Extractions

The extraction of total polyphenols from *Passiflora edulis* was optimized by evaluating different solvent systems and determining the optimal number of successive extractions. Four solvent mixtures were tested: S1 (water), S2 (acetone–water, 7:3), S3 (ethanol–water, 7:3), and S4 (95% ethanol). Lyophilized samples (0.25 g) were extracted with 10 mL of each solvent under constant agitation at room temperature in a sonic bath for 10 min. The supernatant was collected, and additional extractions were performed sequentially on the same solid residue up to five times to assess the effect of repeated extractions on total polyphenol yield. Total polyphenol content (TPC) was measured after each extraction cycle. The quantification was conducted in triplicate using a Biotek-Synergy HT microplate reader, with a shaking sequence of 30 s and a 20 min incubation before measurement.

The optimized conditions were subsequently applied for polyphenol quantification and antioxidant activity evaluation.

### 3.4. Total Polyphenol Determination Using Folin–Ciocalteu

The TPC of *P. edulis* pulp and peel extracts was determined by adapting the Folin–Ciocalteu spectrophotometric method to a microscale format [44]. Approximately 250 mg of dry sample was extracted under optimized conditions using acetone–water (7:3) as the solvent system, applying three successive extractions of 3 mL each. For the assay, 200 µL of distilled water, 15 µL of Folin–Ciocalteu reagent, 30 µL of sample extract, and 50 µL of 20% sodium carbonate solution were added sequentially into each well of a 96-well microplate. A blank was prepared by replacing the extract with solvent. A calibration curve was constructed using aqueous gallic acid solutions in the range of 0 to 0.112 mg/mL. After assembling the reaction mixture, the plate was gently shaken and incubated at 40 °C for 20 min. Subsequently, absorbance was read at 755 nm using a Synergy HT Multi-Detection Microplate Reader (BioTek Instruments, Winooski, VT, USA). TPC values were expressed as milligrams of gallic acid equivalents per 100 g of dry weight (mg GAE/100 g d.w.). All analyses were performed in triplicate.

### 3.5. Determination of Antioxidant Activity by ORAC

The antioxidant capacity of *P. edulis* pulp and peel extracts was assessed using the oxygen radical absorbance capacity (ORAC) method [44], which was adapted to a microplate format. The assay is based on the competition between antioxidant molecules and the fluorescent probe fluorescein for peroxyl radicals generated by the thermal decomposition of AAPH (2,2′-azobis(2-amidinopropane) dihydrochloride). All reactions were performed in 96-well black microplates, using a Synergy HT Multi-Mode Microplate Reader (BioTek Instruments, Winooski, VT, USA), with an excitation/emission filter setting of 485/528 nm.

Each well contained a total volume of 200 µL, which was composed of 120 µL of fluorescein solution (116.7 nM), 20 µL of sample extract or Trolox standard (in 75 mM phosphate buffer, pH 7.4), and 60 µL of freshly prepared AAPH solution (40 mM). After loading the plates, samples were pre-incubated at 37 °C for 15 min. The AAPH solution was added rapidly with a multichannel pipette, and the fluorescence was recorded at 1 min intervals for 80 min.

A standard calibration curve was constructed using Trolox (6.25 to 100 µM), and the results were calculated by comparing the area under the fluorescence decay curve (AUC) of the samples with that of Trolox. Antioxidant capacity was expressed as micromoles of Trolox equivalents per gram of dry weight (µmol TE/g d.w.). All determinations were performed in triplicate.

### 3.6. Bacterial Susceptibility Testing

The antibacterial activity of *P. edulis* extracts was evaluated using the Kirby–Bauer disk diffusion method, following standard microbiological protocols [61]. Four bacterial strains were selected for the assay: two Gram-negative (*Escherichia coli* and *Pseudomonas fluorescens*) and two Gram-positive (*Bacillus subtilis* and *Staphylococcus aureus*) strains. All strains were cultured in Müller–Hinton agar (Oxoid™, Basingstoke, Hampshire, UK) and adjusted to a turbidity equivalent to a 0.5 McFarland standard (approximately 1.5 × 10^8^ CFU/mL) prior to inoculation.

Sterile disks (6 mm diameter) were impregnated with 20 µL of extract solutions at three concentrations: 5 µg, 10 µg, and 20 µg per disk. Disks were placed on the surface of the inoculated agar plates and incubated at 37 °C for 72 h. In a second round of assays, peel extracts were tested at higher concentrations (14 µg, 28 µg, and 56 µg per disk) based on preliminary observations of low activity in Gram-negative strains. Gentamicin (10 µg) was used as a positive control, and the solvent used for extract preparation was included as a negative control.

The results were expressed as percentages relative to the positive control, with both the inhibition zone diameter (IZD) and the relative percentage of inhibition zone diameter (RPIZD) calculated according to the following formula:

$$\%RPIZD=\frac{{IZD}_{of\;extract}-{IZD}_{negative\;control}}{{IZD}_{of\;reference\;antibiotic}}\times 100$$

All experimental measurements were performed in triplicate, and data were expressed as mean values ± standard deviation (SD). The normality of data distributions was assessed using the Shapiro–Wilk test. Since not all datasets met the assumptions of normality and homogeneity of variances, statistical differences between groups were evaluated using the non-parametric Kruskal–Wallis test, followed by Dunn’s post hoc test with Bonferroni correction. Statistical significance was established at *p* < 0.05. Correlation analyses between total polyphenol content (TPC) and antioxidant activity (ORAC values) were performed using Spearman’s correlation coefficient. All statistical analyses were conducted independently for pulp and peel samples using SPSS Statistics version 22 (IBM Corp., Armonk, NY, USA).

## 4. Conclusions

The present study demonstrated that *Passiflora edulis* peel exhibits higher total polyphenol content and antioxidant capacity compared to the pulp across all three ripening stages evaluated. While the concentration of polyphenolic compounds exhibited moderate variations depending on the stage of fruit maturation, the overall antioxidant activity remained stable throughout the ripening process.

Given the stability of antioxidant activity across different ripening stages, *Passiflora edulis* peel can be effectively valorized as a source of bioactive compounds. This flexibility supports its potential use in industrial applications focused on waste utilization and the development of functional products, regardless of the fruit’s maturity state.

The antibacterial assays revealed selective inhibition against Gram-positive bacteria (*Staphylococcus aureus* and *Bacillus subtilis*), supporting the potential of peel extracts as natural antimicrobial agents. No significant inhibition was observed against Gram-negative strains, likely due to structural differences in their cell walls.

The novelty of this study lies in the simultaneous evaluation of antioxidant and antibacterial activities in both peel and pulp across different ripening stages, highlighting underexplored opportunities for valorizing passion fruit by-products within circular economy models.

Future research should aim to incorporate advanced chromatographic techniques, such as HPLC-MS/MS, to further identify and quantify the specific bioactive compounds responsible for the observed biological activities. This would strengthen the development of standardized extracts for use in nutraceutical, pharmaceutical, or food preservation applications.

## Figures and Tables

**Table 1:** Comparison of bioactive compounds in peel (C) and pulp (P) extracts of *P. edulis* at three ripening stages (M1: intermediate; M2: ripe; M3: overripe).

Metabolite	CM1	CM2	CM3	PM1	PM2	PM3
Alkaloids	±	±		+	+	+
Triterpenes	++	++	++++	+	++	++++
Saponins	++++	++++	++++	+	nd	nd
Tannins	±	±	±	nd	nd	nd
Flavonoids	++++	++++	++++	nd	nd	nd
Leucoanthocyanins	nd	nd	++++	nd	nd	nd
Cardiotonic Glycosides	nd	nd	nd	nd	nd	nd
Quinones	nd	nd	nd	nd	nd	nd
Anthocyanins	++++	++++	++++	++++	++++	++++
Coumarins	++++	++++	++++	++++	++++	++++

Qualitative nominal measurement scale (intense/abundant: ++++; moderate: ++; scarce: +; doubtful: ±; not detected: nd).

## Data Availability

The data supporting the findings of this study will be made available upon reasonable request to the corresponding author.

## Abbreviations

The following abbreviations are used in this manuscript:

mg GAE/g d.w.	gallic acid equivalents per gram of dry weight
μmol TE/g d.w.	micromoles of Trolox equivalents per gram of dry weight
IZD	inhibition zone diameter
RPIZD	relative percentage of the inhibition zone diameter
TP	total polyphenol
MIC	minimum inhibitory concentration
LADOQUI	Industrial Chemistry Teaching Laboratory
ORAC	oxygen radical absorbance capacity
AA	antioxidant activity
TPC	total polyphenol content
S1	water
S2	acetone–water (7:3)
S3	ethanol–water (7:3)
S4	95% ethanol
MS	standard deviation
Trolox	6-hydroxy-2,5,7,8-tetramethylchroman-2-carboxylic acid
C	peel
P	pulp
M1	pre-ripe fruit
M2	ripe fruit
M3	overripe fruit
